# Functional and Radiological Outcomes of High Tibial Osteotomy Using the Hemicallotosis Method by Dynamic Axial Fixator in Patients With Medial Compartment Osteoarthritis and Varus Deformity

**DOI:** 10.7759/cureus.111339

**Published:** 2026-06-23

**Authors:** Nilesh Joshi, Kaustubh Zodey, Pallav P Agrawal, Sushil Mankar, Shrikrishna A Rakhunde, Darshan Sharma, Shivani Gaur, Sarvesh S Auradkar, Pranav Datta

**Affiliations:** 1 Orthopaedics and Traumatology, NKP Salve Institute of Medical Sciences & Research Centre and Lata Mangeshkar Hospital, Nagpur, IND; 2 Orthopaedics, NKP Salve Institute of Medical Sciences & Research Centre and Lata Mangeshkar Hospital, Nagpur, IND

**Keywords:** dynamic external fixator, limb deformity, lrs external fixator, mechanical axis deviation, open wedge high tibial osteotomy

## Abstract

Medial compartment osteoarthritis (MCOA) of the knee is a major cause of pain and disability, often associated with varus malalignment. In younger and active patients, high tibial osteotomy (HTO) helps realign the mechanical axis, offloading the diseased medial compartment and delaying the need for arthroplasty. The hemicallotasis technique using a dynamic axial fixator (DAF) offers gradual correction with minimal soft-tissue damage and no internal hardware. This study evaluated the functional and radiological outcomes of HTO using the hemicallotasis method with DAF in patients with MCOA and varus deformity. This longitudinal descriptive study was conducted at a tertiary care teaching hospital from 2021 to 2024. Thirty-seven patients with isolated MCOA and varus deformity underwent medial opening wedge HTO using the hemicallotasis method under image guidance. Those with inflammatory, bicompartmental, or tricompartmental arthritis, or previous fractures around the knee, were excluded. Functional assessment was done using the Oxford Knee Score (OKS) at one, three, and six months postoperatively. Radiological outcomes were evaluated by measuring mechanical axis deviation (MAD). The mean OKS showed progressive improvement, indicating steady functional recovery. Younger patients demonstrated slightly higher baseline and postoperative scores. A strong positive correlation was observed between mechanical alignment correction and functional gain. HTO using the hemicallotasis method with a DAF is an effective, joint-preserving procedure for MCOA with varus deformity. It provides accurate alignment correction, excellent functional improvement, and a low complication rate. This technique offers a valuable option for younger and active patients, maintaining joint function while delaying the need for knee arthroplasty.

## Introduction

Osteoarthritis (OA) is currently one of the most prevalent joint disorders affecting adults worldwide [1]. It is marked by a gradual deterioration of articular cartilage, along with the formation of new bone and, in many cases, synovitis. These changes often lead to pain, reduced joint function, and eventually significant disability.

Symptomatic OA is typically identified by the presence of radiographic changes along with joint pain or stiffness. In the knee, the most common presentation involves degeneration of the articular cartilage, primarily affecting the medial compartment. As the disease progresses, this degeneration can lead to progressive varus deformity. Additionally, individuals with early-stage knee OA often exhibit altered joint loading patterns and gait disturbances [2]. Increased mechanical stress places excessive strain on the cartilage while inflammatory activity contributes to the breakdown and weakening of its structural framework. This can result in cartilage damage under excessive stress.

Mechanically, the causes may be intraarticular, like fractures with irregular chondral surfaces or injuries to the meniscus and cartilage, or extraarticular, like lower limb deformities in the frontal or sagittal planes. In many cases, initial cartilage damage in one compartment of the knee leads to limb malalignment, which in turn exacerbates pressure on the already compromised cartilage [1].

The biomechanical concept of high tibial osteotomy (HTO) in medial compartment OA (MCOA) involves shifting weight-bearing forces from the damaged medial to the lateral compartment [2]. This adjustment helps alleviate pain and slows the progression of the disease. Medial opening wedge HTO (MOWHTO) is an effective treatment for genu varus. Its popularity has grown due to several advantages over closed wedge techniques, including the ability to correct deformity closer to the origin [3]. MOWHTO is easier to implement, lessens the chance of harming the proximal tibiofibular joint and common peroneal nerve. It permits future alterations without sacrificing the original bone stock. MOW osteotomy using the dynamic axial external fixator has recently become popular for varus correction due to its surgical simplicity and precision in achieving correction. Despite these advantages, there have been reports of issues like infections, non-union, hardware migration inside the tibiofemoral joint, osteotomy penetration, and iatrogenic fractures [2]. Nevertheless, these surgeries remain the treatment of choice for young individuals as they can slow down or even prevent degenerative processes or at least delay the need for total knee arthroplasty.

## Materials and methods

This was a longitudinal prospective study conducted in the Department of Orthopedics at the NKP Salve Institute of Medical Sciences & Research Centre and Lata Mangeshkar Hospital, Nagpur, Maharashtra, India, between 2021 and 2024. The study was approved by the NKP Salve Institute of Medical Sciences Institutional Ethical Committee (reference number: 59/2022), and written informed consent was obtained from each participant before enrolment.

Patient selection

Patients included were between the ages of 30 and 60 years, with symptomatic isolated MCOA confirmed by clinical and radiological assessment, varus deformity on full-limb standing scannogram, Kellgren-Lawrence grade II-IV, a willingness to undergo surgery, and to comply with the follow-up schedule.

All patients with lateral or patellofemoral OA, inflammatory arthropathy, prior ipsilateral knee surgery, significant rotational or sagittal plane deformity, and patients not willing for surgery were excluded from the study.

Sample size estimation

Sample size was determined considering the correlation between function and radiological outcome (Hip-Knee-Ankle (HKA) Angle and Oxford Knee Score (OKS)) as the outcome measures. The sample size was calculated using the following formula based on the correlation coefficient: $n = \frac{\left(Z_{1-\beta} + Z_{1-\alpha/2}\right)^2}{\dfrac{r^2}{1-r^2}}$, where, r: correlation coefficient, Z1−α/2: desired confidence level (critical value at significance level α), and 1−β: power of the study. Study-specific values applied included: Correlation coefficient (r) = 0.43, Power (1−β) = 80%, Level of significance (α, two-sided) = 5%. The assumptions were made as for a similar study conducted by Yadav et al. [4].

Assuming 5% losses to follow-up in a three-month period, effected sample size was taken as 37.

Outcome measures

Functional assessment was done using the OKS, which is a validated patient-reported 12-item questionnaire scoring knee pain and function on a scale of 0 (worst) to 48 (best). Assessments were performed preoperatively and at one, three, and six months postoperatively. Permission for the use of OKS was taken from Oxford University Innovation Limited.

**Figure 1:**
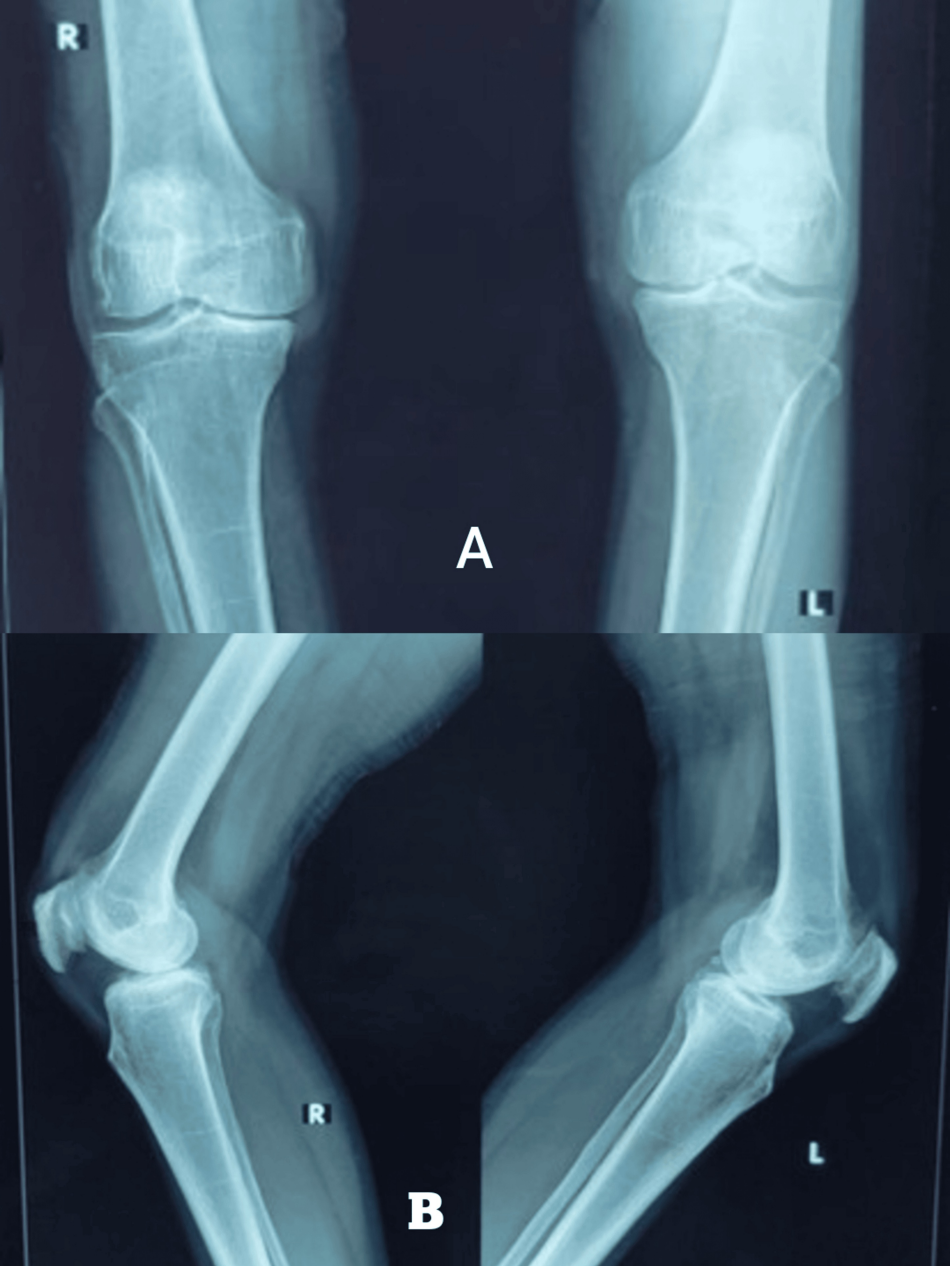
Preoperative radiographs of bilateral knee of a participant (A) Anteroposterior view; (B) Lateral view

Radiological assessment comprised bilateral standing anteroposterior (AP) and lateral radiographs (Figure 1), obtained preoperatively.

**Figure 2:**
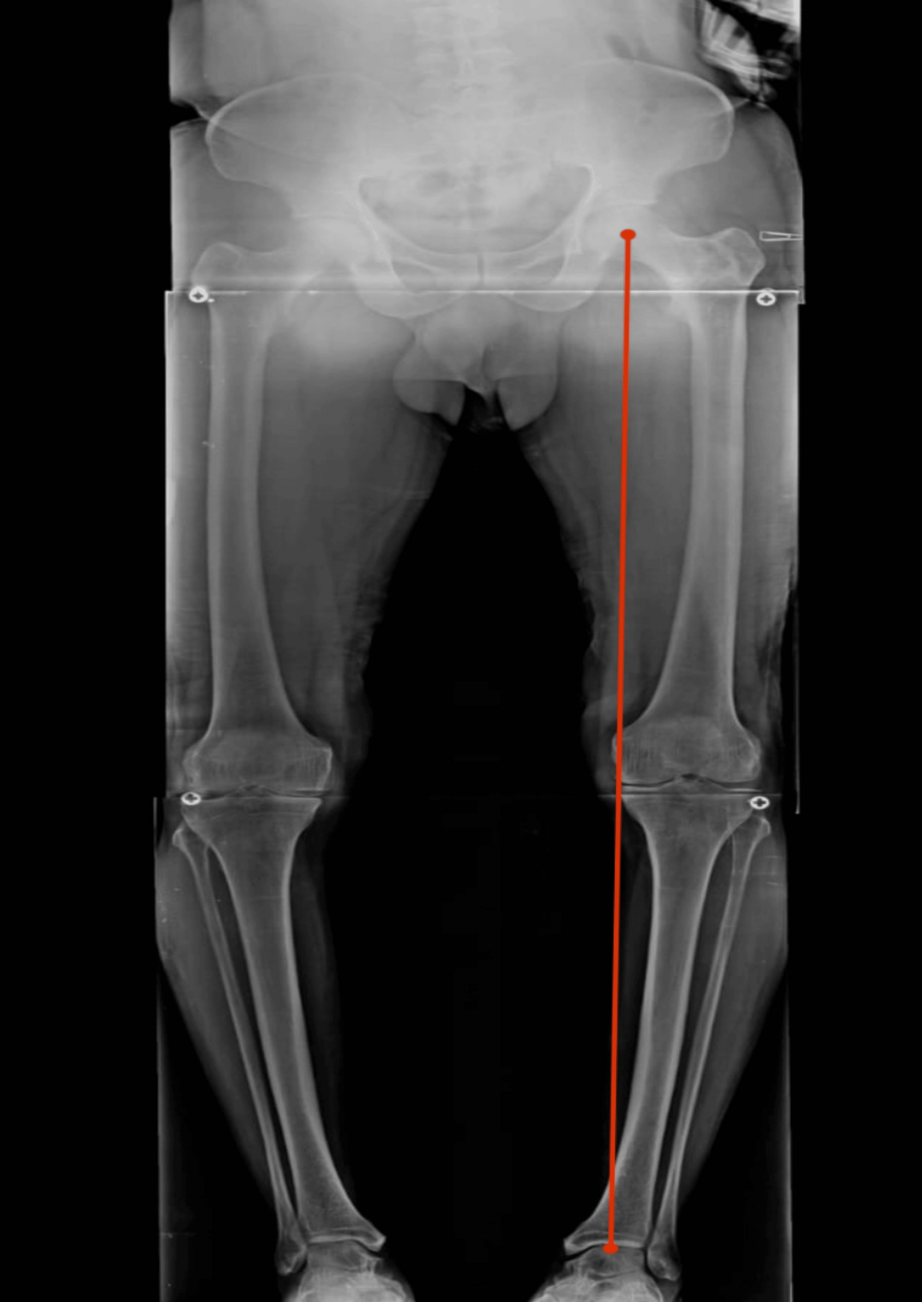
Full length scannogram with mechanical axis of left lower limb.

Full-length scannograms of the patients were taken (Figure 2). Mechanical axis deviation (MAD) is defined as the perpendicular line drawn from the center of the femoral head to the center of the tibial plafond on a digital scannogram. A negative, i.e., medial, MAD value indicates varus malalignment; a positive value indicates valgus alignment.

Surgical technique

**Figure 3:**
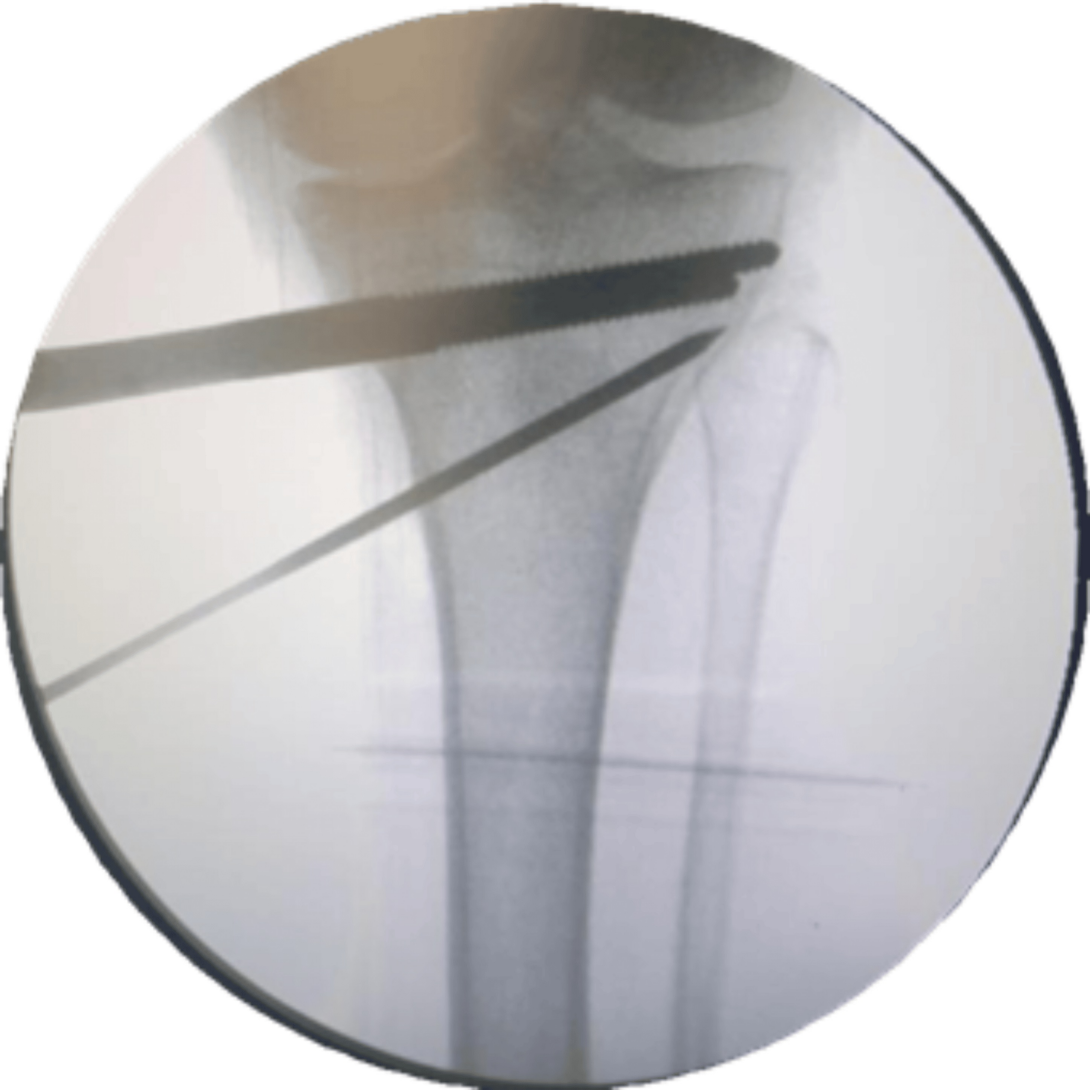
Intraoperative radiograph of two 6 mm tapered Schanz pins placed into proximal tibia parallel to each other and directing at the tip of the fibula.

All procedures were performed under spinal anesthesia. The patient was positioned supine on a radiolucent table. The dynamic axial fixator (DAF) assembly was prepared on the sterile field. After cleaning, painting, and draping the limb, it was brought into a neutral position. Under image guidance, two 6 mm tapered half-pins were placed into the proximal tibia parallel to the joint line after drilling the medial cortex and also parallel to each other and directed at the tip of the fibula, taking care not to perforate the subchondral bone or the posterior cortex (Figure 3).

**Figure 4:**
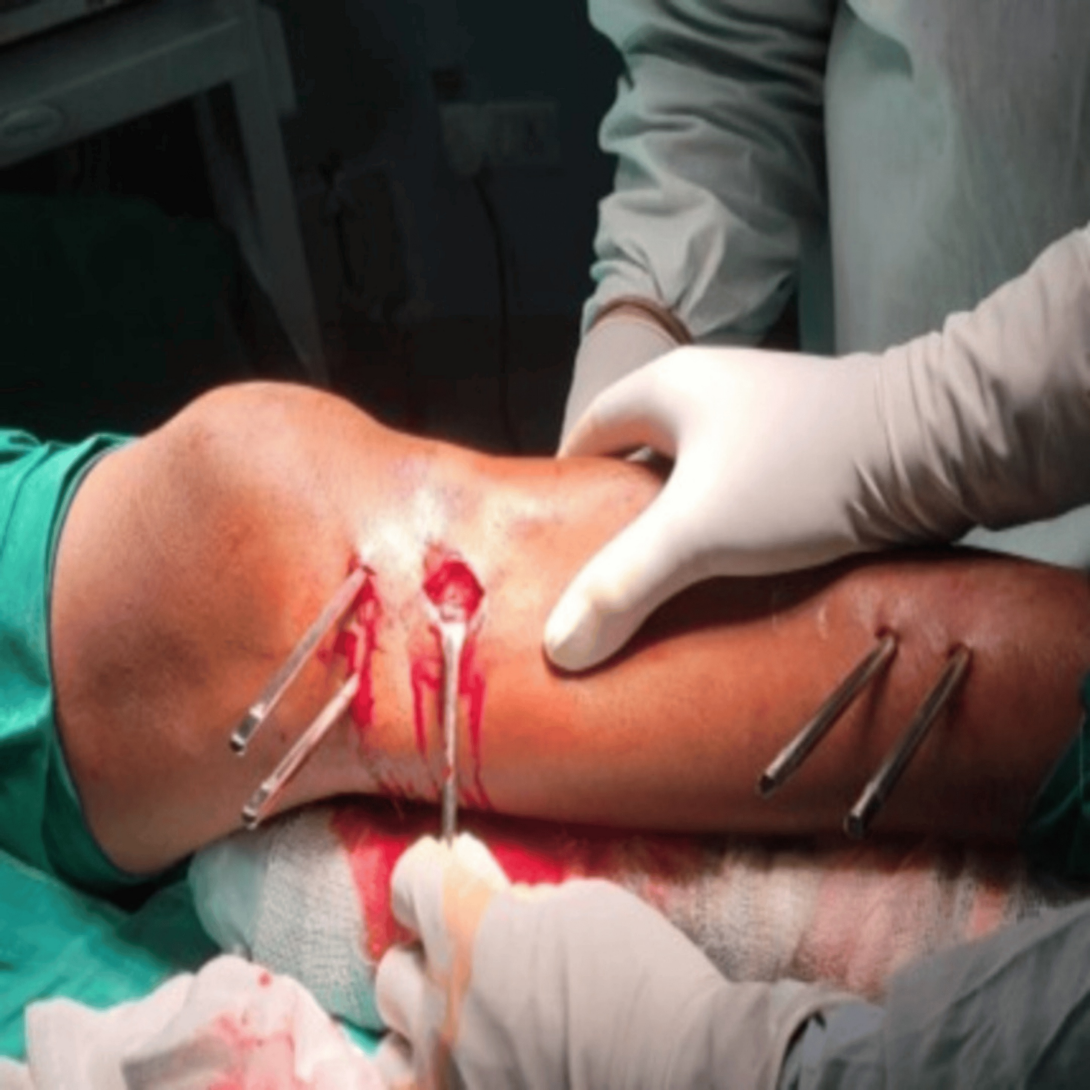
Two tapered half pins placed into the distal tibia and anteromedial incision for osteotomy.

Using the closed frame of the DAF as a guide, two 6 mm tapered half-pins were placed into the distal tibia under fluoroscopic control, maintaining appropriate divergence to maximize bone purchase (Figure 4).

**Figure 5:**
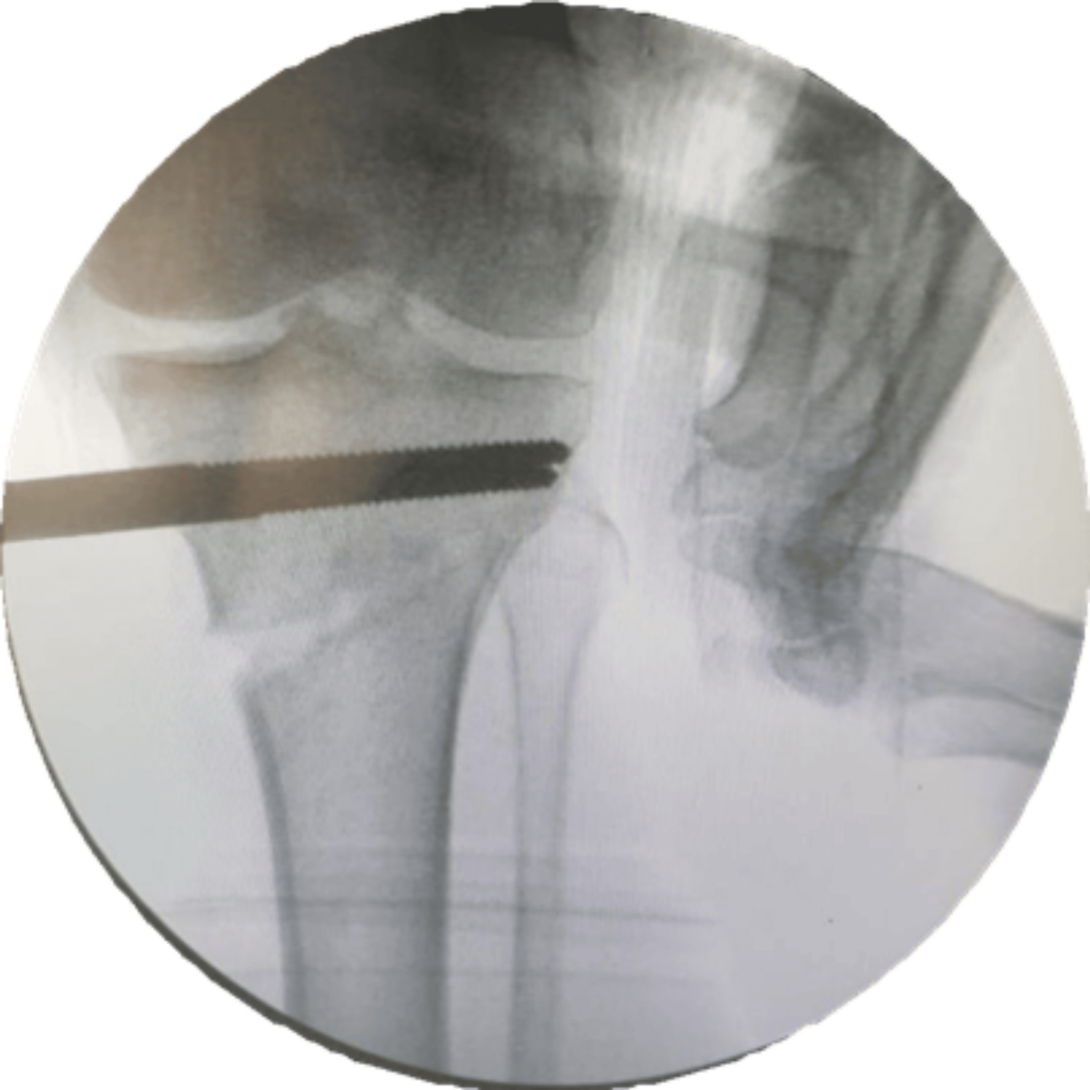
Radiograph image of medial open wedge osteotomy.

Osteotomy was performed through a 2 cm anteromedial incision using a 4.5 mm drill bit at multiple points along a vertical line, approximately 3-4 cm distal to the joint line, preserving the lateral cortex. Corticotomy was completed with sharp osteotomes (Figure 5).

**Figure 6:**
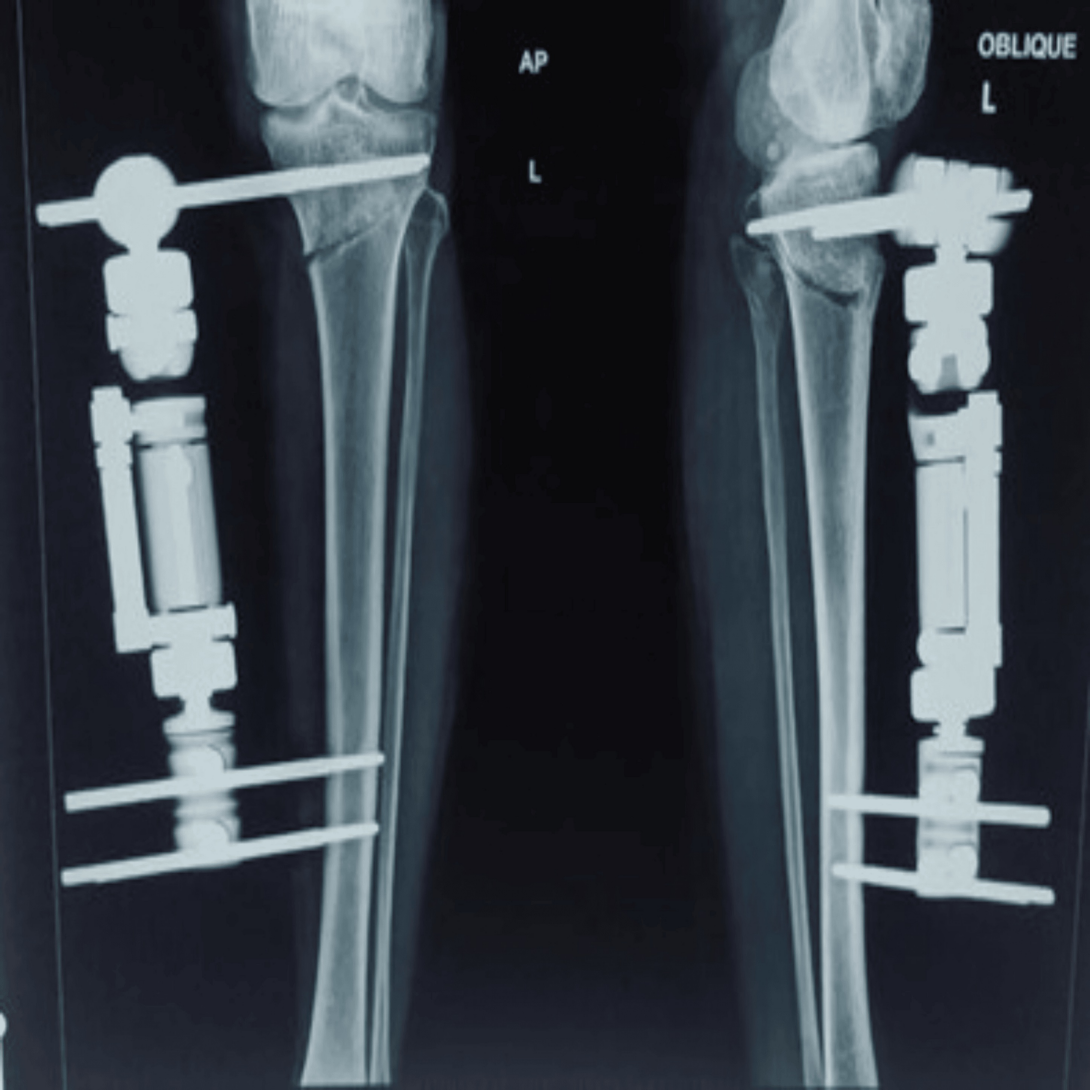
Immediate postoperative radiograph.

The fixator frame was then assembled, the wound was closed, and sterile dressings were applied. An immediate postoperative radiograph confirmed satisfactory osteotomy placement (Figure 6).

**Figure 7:**
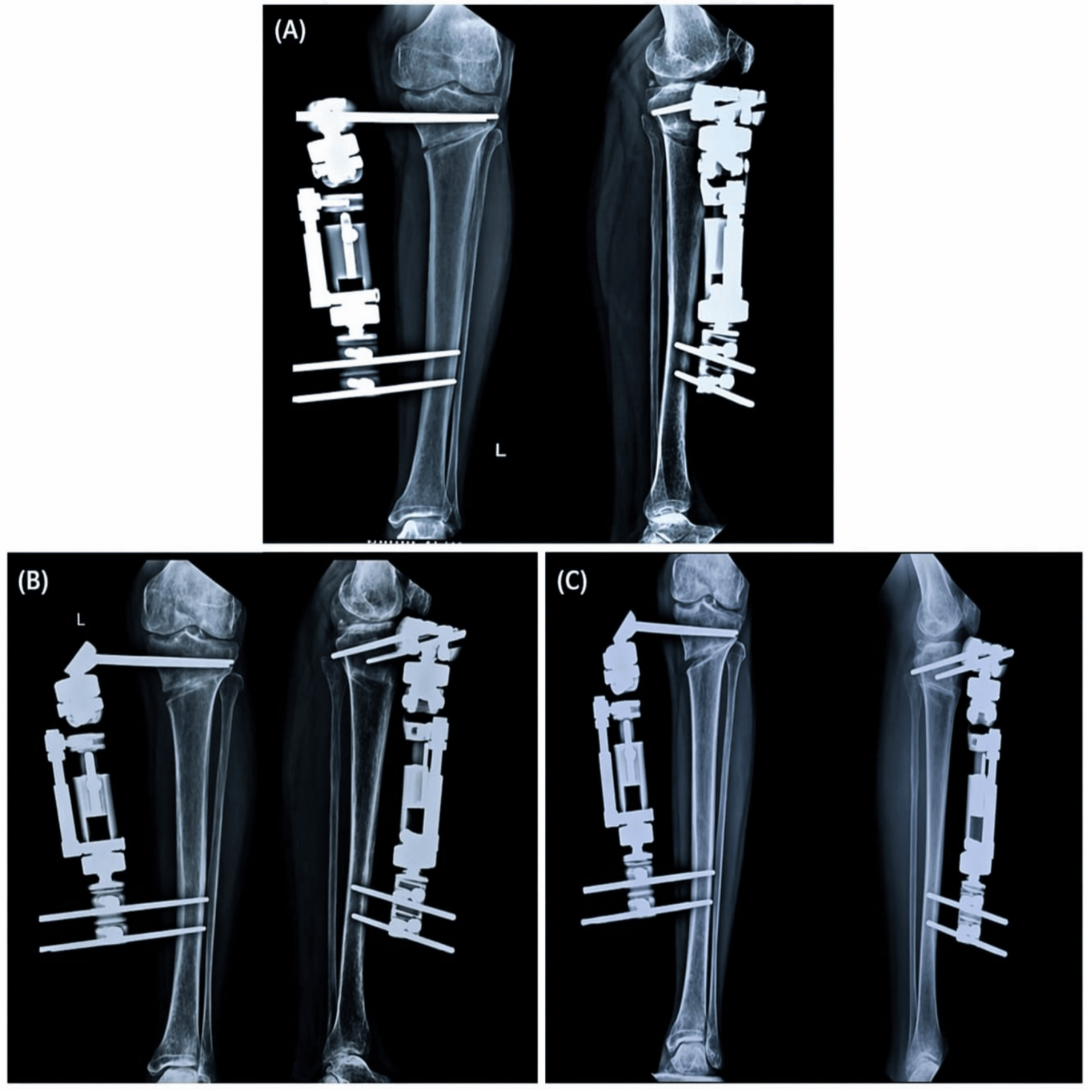
Follow-up radiographs. (A) at one month; (B) at three months; (C) at six months

Distraction was commenced on postoperative day 5 at a rate of 1 mm per day until the required correction was achieved, followed by serial radiographic assessment and clinical examination (Figure 7). 

**Figure 8:**
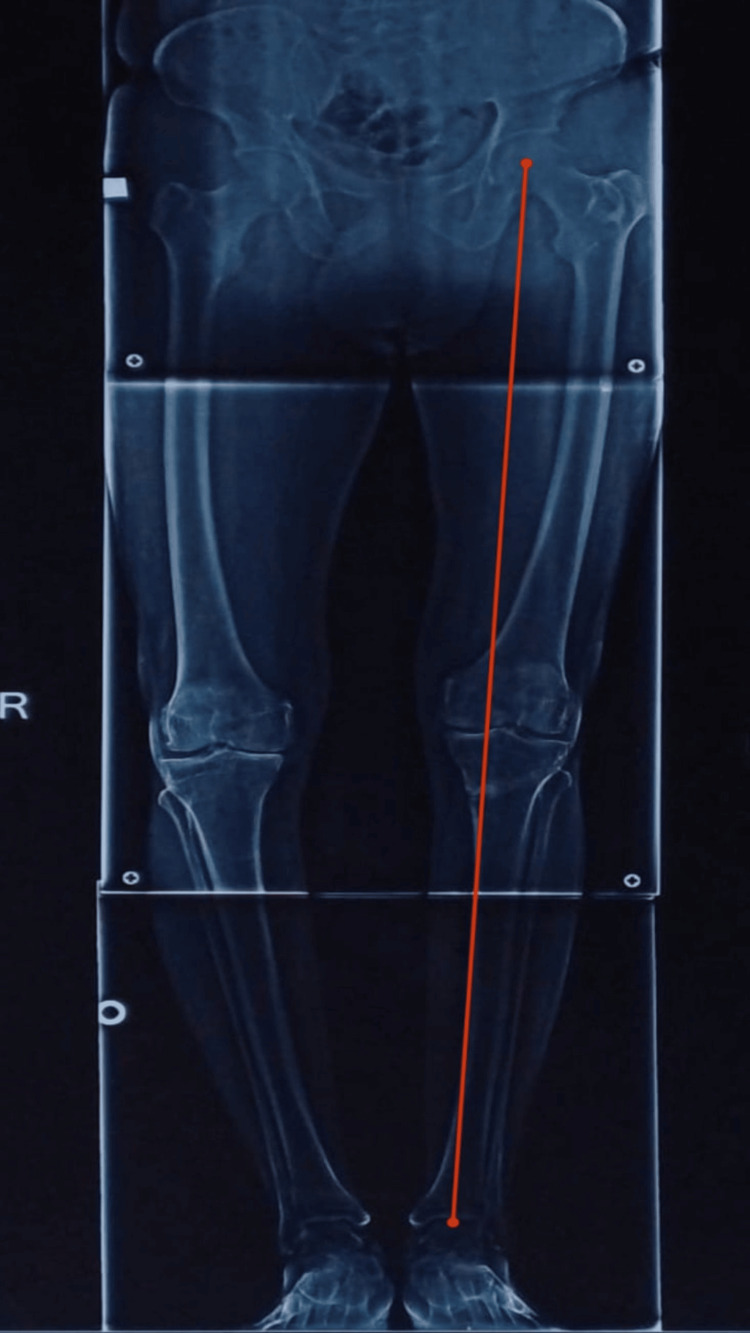
Scannogram post removal of DAF and corrected mechanical axis of left lower limb. DAF: dynamic axial fixator

Patients were permitted to partially bear weight with crutch support from the first postoperative day. Pin-site care was performed by the patient with daily chlorhexidine gauze dressings. The fixator was removed in an outpatient setting under local anesthesia, once radiological consolidation of the regenerate was confirmed (Figure 8).

Statistical analysis

Data were entered and analyzed using IBM SPSS Statistics for Windows, version 25.0 (IBM Corp., Armonk, New York, United States). Continuous variables are expressed as mean ± standard deviation (SD). The Wilcoxon signed-rank test was used for paired pre-post comparisons of OKS and MAD. Spearman's correlation coefficient was used to assess associations between MAD and OKS. Categorical variables are presented as frequencies and percentages. A p-value of < 0.05 was considered statistically significant.

## Results

Demographic characteristics

Thirty-seven patients were included and completed a six-month follow-up. The mean age was 48.4 ± 5.1 years; 27.0% (n = 10) were aged 37-45 years and 73.0% (n = 27) were aged 46-55 years. There were 20 (54.1%) female patients and 17 (45.9%) male patients. Left-sided involvement was slightly more common (54.1%, n = 20) than right-sided (45.9%, n = 17) (Table 1).

**Table 1:** Baseline demographic and preoperative characteristics (N = 37)

Variable	Category	Value
Age group (years), n (%)	37-45	10 (27.0%)
	46-55	27 (73.0%)
Mean age (years), mean±SD		48.4 ± 5.1
Gender, n (%)	Male	17 (45.9%)
	Female	20 (54.1%)
Side, n (%)	Left	20 (54.1%)
	Right	17 (45.9%)
Preoperative mechanical axis deviation, mean±SD		−3.45 ± 13.62
Preoperative Oxford Knee Score, mean±SD		21.56 ± 5.76

Radiological outcomes

Mean preoperative MAD was −3.45 ± 13.62, showing overall varus malalignment. Following hemicallotasis correction, mean MAD at six months improved significantly to 48.16 ± 4.58 (p < 0.0001), representing a mean correction of 51.61. Younger patients (37-45 years) exhibited a mean preoperative MAD of 7.0 ± 9.67, while older patients (46-55 years) showed −6.48 ± 13.23 (p = 0.002), indicating greater varus malalignment in the older cohort (Table 2).

**Table 2:** Pre- and postoperative mechanical axis deviation (MAD) comparison.

Parameter	Preoperative value	Six-month posterative value	p-value
Mean mechanical axis deviation (MAD)	−3.45 ± 13.62	48.16 ± 4.58	< 0.0001
Range	−29 to +28	+38 to +55	—
Mean correction	—	51.61	—

Functional outcomes (OKS)

OKS showed a statistically significant and clinically progressive improvement across all follow-ups. Mean OKS at baseline was 21.56 ± 5.76. At one month, scores rose to 25.00 ± 5.80 (p = 0.012 versus preoperative). At three months, scores improved further to 29.62 ± 5.74 (p < 0.0001). By six months, mean OKS reached 38.46 ± 5.01 (p < 0.0001 versus baseline), representing an absolute mean improvement of 16.9 points. Spearman's correlation demonstrated a significant positive association between postoperative MAD improvement and six-month OKS (ρ = 0.68, p < 0.001), confirming that better mechanical axis correction correlated with superior functional outcomes (Table 3).

**Table 3:** Serial Oxford Knee Score (OKS) outcomes.

Time Point	Mean OKS	SD	Range	p-value
Preoperative	21.56	5.76	14–32	-
1 Month	25.00	5.80	17–34	0.012
3 Months	29.62	5.74	19–38	< 0.0001
6 Months	38.46	5.01	29–45	< 0.0001

Subgroup Analysis

Younger patients (37-45 years) demonstrated a higher mean preoperative OKS of 26.22 ± 4.57 compared with the 46-55-year group (19.96 ± 5.40; p = 0.002), indicating better baseline functional reserve in the younger cohort. Both subgroups showed significant postoperative improvement, though younger patients achieved higher absolute postoperative OKS values, consistent with their higher pre-morbid functional level.

Complications

The overall complication rate was low. Of 37 patients, 94.6% (n = 35) experienced no complications. Superficial pin-tract infection occurred in 5.4% (n = 2) and was successfully managed with a 10-day course of oral antibiotics and enhanced pin-site hygiene, without requiring fixator removal. No deep infections, osteomyelitis, septic arthritis, non-union, malunion, peroneal nerve palsy, or hardware failure were documented during the study period.

## Discussion

In patients with MCOA and varus deformity, the study examines the functional and radiological results of HTO using hemicallostasis with a DAF. This approach aims to address the challenges of knee alignment and improve patient outcomes by providing precise correction without the need for permanent implants.

In the current study, the majority of cases (72.97%) were in the 46-55 age group, with a smaller proportion (27.03%) in the 30-45 age group. Robinson et al. found a mean age of 48.7 years [5], which was close to the younger end of our study's age range. Similarly, Hernándezet al. reported a mean age of 50.5 years [6], which was similar to the present study. Overall, the present study shows a higher proportion of cases in the 46-55 age range; the ages reported in other studies are somewhat consistent with or slightly older than this predominant age group. Contrary to the findings of our study, Klinger et al. reported a median age of 56 years (33-66 years), indicating a slightly older population [7].

The gender distribution in the present study revealed that female patients accounted for 54.05% of the cases, while males comprised 45.95%. Similar findings were observed in a study conducted by Kumar et al., who reported that 95% of their patients were female [8]. The results were also in agreement with the study carried out by Gupta et al. [9]. In contrast, Zhang et al. reported a total of 32 patients (20 male and 12 female), indicating a lower proportion of female patients (37.5%) [10].

In the present study, 54.05% of cases affected the left knee while 45.95% affected the right knee, indicating a slight predominance of left-sided involvement. This contrasts with findings from Kumar et al. [8], who reported an equal distribution between right and left knees. Another study by Nikose et al. found that 76.93% of the knees affected were right, with only 23.07% being left [3]. Young, active people with OA in their knees were shown to have a higher prevalence on their dominant side, and there was no difference in limb length following surgery.

In the present study, preoperative MAD had a mean of -3.45 ±13.62, which improved significantly to 48.16 ± 4.58 after six months, demonstrating the effectiveness of the intervention. Comparatively, Yadav et al. reported a mean preoperative MAD of 14 ± 4 and postoperative MAD of 30 ± 12 [4], showing substantial improvement similar to our results. Bachhal et al. aimed for a 2-8 degree valgus correction in 37 knees, achieving desired alignment in 31 knees (84%), with under-correction in five knees and over-correction in one [11]. Kapila et al. aimed for a 6-8 degree valgus correction in 30 knees but achieved only 5-7 degrees in 60% of their cases [12]. While our study achieved a notable improvement in MAD, the rates of desired alignment and correction are comparable to those reported by Yadav et al. [4] and Bachhal et al. [11], but somewhat less favorable than the 60% success rate of Kapila et al. [12].

The OKS across 37 participants in the current study varied from 14 to 32, with most scores falling between 14 and 29. Scores of 15 and 16 were the most frequently observed, each accounting for 13.51% of the cases. Lower scores, such as 14, 17, and 18, were less common, as were higher scores, such as 25 and 28. The distribution indicates a broad range of outcomes with no single score being predominant. The mean OKS in our study showed significant improvement over time, with scores of 25 ± 5.80 at one month (p < 0.0001), increasing to 29.62 ± 5.74 at three months and reaching 38.46 ± 5.01 by six months. Preoperative scores averaged 20 ± 9, while postoperative scores improved to 42 ± 4 at the end of six months, indicating notable progress. Abdelkader and Mustafa reported a preoperative mean OKS of 17.1 ± 0.24 and a postoperative mean of 44.5 ± 0.28, reflecting significant improvement [13], which was parallel with this study's findings. Gupta found statistically significant improvements in OKS in both the Orthofix and Tomofix groups, with p-values of 0.004 and 0.002, respectively [9]. Similarly, Bachhal et al. reported a significant improvement from a mean preoperative score of 19.11 to a mean postoperative score of 43.05 (p < 0.01) [11].

In the current study, 94.59% of patients experienced no complications, while 5.41% had superficial pin tract infections. The chi-square value indicates a statistically significant difference, highlighting the rarity of complications. In contrast, Yadav et al. found that pin tract infections were the most frequent complication affecting 10% of patients with additional cases of septic arthritis (3%) and early union of the osteotomy (3%) [4]. Bode et al. revealed that 25% of cases developed minor pin tract infections, and one patient experienced nonunion [14]. McClelland et al. observed a higher incidence with 37% of patients developing pin tract infections; four knees were converted to TKA, and one case involving a proximal pin-site infection resulting in an infected TKA [15].

The distribution of preoperative OKS by age group in the present investigation shows that patients aged 37-45 had a mean preoperative score of 26.22 ± 4.57, while those aged 46-55 had a lower mean score of 19.96 ± 5.40. The p-value indicates a statistically significant difference, suggesting that younger patients (37-45 years) had higher preoperative scores compared to their older counterparts (46-55 years). A similar finding was obtained in the study carried out by Vora et al., in which the most significant improvement in OKS occurred in females aged 51-60 years, whose preoperative OKS improved from 22.6 to 43.4 postoperatively [16]. Conversely, females aged ≥60 years showed less improvement, though it was still significant.

The mean OKS significantly enhanced from 21.56 ± 5.76 preoperatively to 25 ± 5.80 at one month (p = 0.012) and continued to rise to 29.62 ± 5.74 at three months and 38.46 ± 5.01 at six months (p< 0.0001). Ravidas et al. noted a shift from 6.2% preoperative scores to 80% postoperatively [17]. Gupta also observed significant improvements with preoperative OKS of 44.5 ± 0.28 and Western Ontario and McMaster Universities Arthritis Index (WOMAC) score of 79.5 ± 0.3, improving to 17.1 ± 0.24 and 45.1 ± 0.3, respectively [9]. These findings align with the current study, which confirms that improvements in the HKA angle are positively correlated with better functional outcomes.

The preoperative MAD had a mean of 21.57, which significantly differs from the postoperative MAD at six months, with a mean of 48.16. This indicates a notable improvement in mechanical axis alignment following correction, which was parallel with the study conducted by Yadav et al. [4]. The MAD significantly enhanced from a preoperative mean of 3.46 to a postoperative mean of 48.16. This was in agreement with the research by Bayam et al., where the preoperative MAD score was -3.7, and it significantly improved to 33.6 postoperatively [18].

As the MAD improves, there is a corresponding improvement in the OKS. This correlation indicates that the alignment of the mechanical axis is a significant predictor of the patient's functional outcome. The mean MAD increased from −3.45± 13.62 preoperatively to 48.16 ± 4.57 postoperatively, a mean difference of 51.61. Similarly, the OKS rose from 21.56 ± 5.76 to 38.46 ± 5.01 with a mean difference of 16.9. The average knee score prior to surgery was 42.96 ± 8.39, and it increased to 89.15 ± 8.26 after surgery. At 47.52 ± 8.15, the functional knee score was higher, which was improved to 78.56 ±6.65. In comparison, Nikose et al. reported an improvement in the mechanical axis from 3.26% preoperatively to 61.81% post correction [3]. Pande et al. noted a mean preoperative OKS of 28.7, improving to 35.4 postoperatively (p = 0.0142) [19]. Additionally, according to Robinson et al., 67% of their patients had documented pin-site infections, and the survival rate of high tibial osteotomies was 88.9% at a median follow-up of 19 months [5].

Study limitations

The relatively small sample size of the study might restrict the ability to apply its findings to a larger population. The longitudinal nature of the study may have limited the follow-up period, potentially affecting the assessment of long-term outcomes and the durability of the results. Only coronal plane deformities were included; other deformities like rotational and sagittal plane were excluded. Variations in surgical technique or the skill level of different surgeons could introduce variability in outcomes, affecting the consistency of the results.

## Conclusions

Varus knee deformity in patients is frequently observed in everyday outpatient departments and affects the medial compartment of the knee joint. Various intra-articular and extra-articular HTOs have been documented as effective methods for correcting this deformity. These can be done using plates or external fixators. In our study, we treated idiopathic varus deformity by HTO using a dynamic external fixator with good postoperative outcomes. HTO using a DAF is a safe, effective, and minimally invasive method for unloading the medial compartment, improving pain and function of the knee. This technique offers precise and adjustable correction, achieves desired outcomes with good healing rates, and avoids the need for bone grafting and permanent implants. Optimal results are achieved with accurate preoperative planning and a skilled surgical approach.

## References

[1] Liu X, Chen Z, Gao Y, Zhang J, Jin Z (2019). High tibial osteotomy: review of techniques and biomechanics. J Healthc Eng.

[2] Guilak F (2011). Biomechanical factors in osteoarthritis. Best Pract Res Clin Rheumatol.

[3] Nikose SS, Nikose D, Kekatpure AL, Jain S, Saoji K, Reddy SM (2020). Impact of medial open-wedge high tibial osteotomy for medial compartment osteoarthritis of the knee. World J Orthop.

[4] Yadav AK, Parihar M, Gawhale S (2021). Functional outcome of high tibial osteotomy in patients with medial compartment osteoarthritis using dynamic axial fixator. Orthop J Sports Med.

[5] Robinson PM, Papanna MC, Somanchi BV, Khan SA (2011). High tibial osteotomy in medial compartment osteoarthritis and varus deformity using the Taylor spatial frame: early results. Strategies Trauma Limb Reconstr.

[6] Hernández MB, Moreno FL, Galán CR, Cabrera JC (2023). Clinical-functional and radiographic outcomes of medial valgus-producing tibial osteotomy. Rev Esp Artrosc Cir Articul En.

[7] Klinger HM, Lorenz F, Härer T (2001). Open wedge tibial osteotomy by hemicallotasis for medial compartment osteoarthritis. Arch Orthop Trauma Surg.

[8] Kumar TK, Issac A, Pai PK, Rajasubramanya P (2021). Early outcomes of high tibial osteotomy using dynamic axial external fixator in medial compartment osteoarthritis of knee. Paripex Indian J Res.

[9] Gupta AK, Mukherjee D, Kumar S (2024). Clinical outcome of high tibial osteotomy by hemicallotasis using a dynamic axial fixator on 52 knees. J Orthopaed Traumatol Rehab.

[10] Zhang Z, Tao H, Zhao Y, Xiang W, Cao H, Tao F (2023). High tibial osteotomy improves balance control in patients with knee osteoarthritis and a varus deformity. J Orthop Surg Res.

[11] Bachhal V, Sankhala SS, Jindal N, Dhillon MS (2011). High tibial osteotomy with a dynamic axial fixator: precision in achieving alignment. J Bone Joint Surg Br.

[12] Kapila R, Sharma PK, Chugh A, Singh R (2015). Management of osteoarthritis knee bygraduated open wedge high tibial osteotomy in 40-60 years age group usinglimb reconstruction system: a clinical study. J Clin Diagn Res.

[13] Abdelkader MA, Mostafa AG (2021). Opening wedge high tibial osteotomy in medial compartment osteoarthritis using dynamic axial fixator. Egyptian Orthop J.

[14] Bode G, von Heyden J, Pestka J, Schmal H, Salzmann G, Südkamp N, Niemeyer P (2015). Prospective 5-year survival rate data following open-wedge valgus high tibial osteotomy. Knee Surg Sports Traumatol Arthrosc.

[15] McClelland D, Barlow D, Moores TS, Wynn-Jones C, Griffiths D, Ogrodnik PJ, Thomas PB (2016). Medium- and long-term results of high tibial osteotomy using Garches external fixator and gait analysis for dynamic correction in varus osteoarthritis of the knee. Bone Joint J.

[16] Vora P, Shah R, Desai B, Taneja S, Pandit J, Amin P (2018). Mid-term results of high tibial osteotomy for medial compartment arthritis using hemicallostasis by dynamic external fixator. Int J Orthop.

[17] Ravidas S, Palak J, Manjhi LB (2019). Functional and radiological outcome of high tibial osteotomy in osteoarthritis patients with varus knee. International Journal of Orthopaedics.

[18] Bayam L, Erdem M, Gülabi D, Erdem AC, Uyar AÇ, Kochai A (2020). Clinical and radiological outcomes of high tibial osteotomy with combined fixator-assisted nailing and subtubercle tibial osteotomy. Acta Orthop Traumatol Turc.

[19] Pande H, Thakur K, Dubey R, Singh C (2021). Changes in lower limb alignment and their effect on the functional outcome after treatment of varus degenerative OA knee by hemicallotasis using modular dynamic HTO fixator. J Clin Orthop Trauma.

